# Supplementary material for: “We couldn’t think in the box if we tried. We can’t even find the damn box”: A qualitative study of the lived experiences of autistic adults and relatives of autistic adults
Source: PLoS One. 2022 Mar 14;17(3):e0264932. doi: 10.1371/journal.pone.0264932 (PMC8920184; doi:10.1371/journal.pone.0264932)
Supplement: Attachment — Submitted filename: Response-adults autism.docx [file pone.0264932.s001.docx]

**Autism Adults paper – PLOS One Reviewer’s comments**

|  | **Reviewer comments** | **Response/ actions** |
| --- | --- | --- |
| R1 | The authors presented a large-scale, qualitative study to examine the life experiences of autistic adults living in the UK. Using thematic analysis, the authors came with six key themes reflecting autistic adults’ experiences in relationships, education, employment, access to services, sense of belonging, and general satisfaction with life. Autistic adults’ accounts were complemented with the analysis of the accounts provided by the relatives of autistic adults that were not able to directly participate in the study. The study is of high quality and covers an important though understudied area of autistic adults’ everyday life. However, some major issues must be addressed before the manuscript will be suitable for publication. | We thank R1 for the assessment that the study is of high quality and addresses and important and understudied topic.  We believe that we have addressed the major issues raised by the reviewers in the responses that follow.  We wish to clarify that relatives’ accounts were ***not*** included in the study solely for the purpose of reporting ***on behalf of autistic adults who were not able to participate in the study directly***. Thank you for highlighting this issue. On re-reading the manuscript, it is clear that our presentation of the rationale for the relative interviews has caused confusion. The relatives were recruited from the ASC-UK cohort. The details of the recruitment procedures for relatives both to the ASC-UK cohort and for this qualitative study has now been revised – see below. |
|  | 1) My main concern when reading the manuscript was related to the adequate representation of the autistic population that was studied. The authors aimed to include a diverse sample of individuals with a wide range of intellectual abilities and this was a rationale to include relatives of those adults who would be unable to express themselves in an interview. However, only six out of 16 relatives reported about individuals with learning disability. This raises a question, what were the reasons for no direct participation of the rest of this sample? Moreover, in my opinion, the experience of individuals with learning disability (or/and more severe autism symptoms) was not highlighted in the paper. This may lead to overgeneralization of the findings and suggest an overly unitary view of the autistic adults’ experience, which may not necessarily be true. Perhaps, relatives of autistic adults with learning disability spoke about experiences that were not in line with the key themes raised by individuals with no such disability (e.g., being dependent in self-care, living in a residential center, or receiving special education). According to the authors’ statement – these relatives’ accounts (not in line with the main themes) were purposively not included in the paper. In fact, autistic participants and relatives of autistic adults are not only two kinds of informants, but they report about two different populations. However, only the first population, of more able adults (i.e., direct participants), served as a basis for identifying the key themes. To sum up, I feel that the findings regarding people with learning disability or more severe autism symptoms (reported by the relatives) should be reflected more in the results, or this subsample should not be included at all to avoid the false notion of representing individuals who were not, in fact, fully represented. | We agree with the reviewer that the findings reported in the paper do not fully represent the experiences of individuals with learning disability or more severe symptoms related to autism.  Certainly the rationale for the recruitment of relatives of autistic adults, to the ASC-UK Cohort sample, was to ‘ *facilitate the inclusion in ongoing research, of the perspective of adults on the autism spectrum who are unable to report about their lives’*. However the broader aims of the cohort was to learn about the experiences of autistic adults of all abilities and the experiences of relatives of autistic adults of all abilities. It was not an inclusion criteria that relatives were only eligible to join the cohort if they were reporting on an autistic adult who was unable to report about their own lives. We have amended the statement in the Methods Section (‘participants’, **Lines 136-140**) as follows:  *In the ASC-UK study, relatives of autistic adults were included to facilitate the inclusion in ongoing research, of the perspective of adults on the autism spectrum across the ability range including those who were unable to report about their lives due to intellectual disability or for other reasons, in accordance with the NHS Constitution (Crown copyright 2015).*  In the qualitative study, relatives of autistic adults were recruited and interviewed about both (1) the lives of their autistic relatives and (2) their own experiences as a relative of an autistic adult. For further clarification we have as requested by R1, included the interview topic guides (for both the autistic adults’ and the relatives’ interviews) as additional material (Appendices 1 & 2). In this manuscript we are including the information from the relatives’ interviews about the experiences of the autistic adults. The relatives’ accounts of their own experiences supporting an autistic relative through different life stages and events, will be reported in a separate manuscript currently in preparation.  To clarify regarding R1’s query relating to overlapping and/or the possibility of missing themes between the two datasets:  The two datasets were analysed separately, and checked for overlapping themes. The themes from this separate analysis that related to the *relatives’ observations of experiences of the adult* aligned with the themes identified in the analysis of the adults’ interviews, and no additional themes relating to their adults’ experiences were identified to add to the themes identified in the adult interviews dataset. Indeed, as representing direct experiences, the adults analysis provided additional depth and detail, relative to the corresponding theme descriptions from the relatives’ dataset (as might have be expected). The key driver here was that the voices of the autistic adults should guide the analysis. The contribution to this paper from the relatives’ dataset should be understood therefore as ‘supplementing’ the adult dataset driven analysis and reporting of themes relating to the experiences of autistic adults, rather than as extending those themes through representation of autistic adults with different sets of challenges and needs. Whilst not fully representative of all autistic adults, we feel we have *added some diversity* of perspectives by including data from relatives interviews about a younger sample (Mean age of 19 years; compared with mean age of 41 years for autistic adults sample) and including some with LD, although not sufficient to be considered representative. To go further however in addressing these concerns, we have now added additional data into both of the participants’ characteristics tables (extracted from the ASC-UK Cohort database) relating to learning disability diagnoses (see columns headed ‘autism spectrum diagnosis and LD).  Therefore we have **removed** the following statement:  *The inclusion of views from relatives of autistic adults who lack capacity [25] is a key feature of this study, since published studies concerning autistic adults most commonly focus on ‘high functioning’ participants [26].*  And have amended the manuscript to make these limitations to representation clear:  In the Methods section (participants, lines 140-148):  *In the qualitative study, relatives were interviewed both about their autistic adult relative (reported in this paper), and about their experiences of supporting an autistic relative (the latter focus not reported here). Since published studies concerning autistic adults most commonly focus on ‘high functioning’ participants [36], the inclusion of accounts from relatives of autistic adults provided an opportunity to extend the focus of the qualitative study to include the experiences of an additional sample of autistic adults some of whom were not able to report about their own lives [26]. Whilst this inclusion extends the size and general diversity of sample on whom we report, broader representation of autistic individuals with learning disability is not assumed.*  And in the ‘analysis’ section (lines 204-205; 210-220 we have made the process of integrating the two datasets clearer by adding:  *The separate analyses were mapped against each other, with particular attention to the themes under ‘experiences of the adult’. A set of general themes under this category was common to both datasets. Although description of the constitutent themes was more detailed in the adults’ dataset, there were no additional themes to contribute to the analysis from the relatives’ dataset. Therefore, from the analysis of relatives’ interviews, data corresponding to relatives’s reports of the experiences of the autistic adults are included within the central themes identified in the adults’ dataset. This enabled us to retain the focus on the perspectives from the accounts of autistic adults, but with additional opportunities to consider the reports of a different sample of autistic adult experiences provided by family members who may also provide support and take on advocacy roles for their autistic adult relatives. The experiences of the relatives themselves will be reported elsewhere (Hamilton et al , in preparation).*  The revised wording now more accurately reflects the role of the relatives’ data in this paper, and we hope, addresses R1’s questions about the analysis process. We have also acknowledged in the discussion the likely limitations for the generalisability of our findings to autistic individuals with learning disability (line 811). |
|  | 2) The introduction sometimes lacked coherence and structure. For example, paragraph two starts with the topic of access to services which seems a bit out of place there and then moves to a different topic (lack of research about life experiences, starting from line 65) without a visible link between the two. The introduction should include a short review of existing literature (especially qualitative ones) related to the topic. Stating that this literature is scarce does not seem sufficient. | We have revised the introduction substantially, as recommended to improve structure and coherence, and have provided a brief review of the existing literature including references recommended by both reviewers.  The structure is now as follows:   1. Brief paragraph of context – increasing need to understand autism in adulthood; importance for the community, research, policy and service provision 2. Account of general literature about autism in adulthood, and what the priorities are/ should be 3. Summary of existing qualitative studies on key topics and/or with particular groups; and what remains as gap to address 4. Paragraph outlining how improved priorising/ knowledge might support better service provision 5. Context of the study, the qualitative paper reported, and its contribution |
|  | 3) I lacked some important characteristics in the description of the participants, notably their education (commonly participants in this kind of research have higher educational attainment than the respective population). It would also be interesting to know if the participants had any co-occurring psychiatric or developmental conditions (the authors mention them later in the results but here more systematic data would be useful). Lastly, the authors included a geographical location in their purposive sampling so it would be good to report at least the size of the residential area where the participants lived (e.g., small/medium/big city, countryside). As the authors aim for the wide representation of the autistic adult population, I think this kind of information is important to provide. | We agree with the reviewer that additional participant data are helpful for understanding the characteristics and representativeness of our sample. On joining the ASC-UK cohort individuals were asked a large number of questions, that included co-ocurring mental health diagnoses, learning disability diagnoses, and educational attainment. We have gone back to the cohort database to extract and now report data for these characteristics as requested (Tables 1 and 2).  As also requested, this information (and other from the table) is also summarised in the text description accompanying the tables, to allow easier assessment of sample representativeness.  Lastly, we have revised the methods (lines 173-175) and results (Lines 240-244; 266) to give more detail of participants’ geographical locations. |
|  | 4) In the beginning of theme 2, the authors write: “For some participants, relationships with partners were described as challenging” but there is no elaboration on that. It would be interesting to read more about romantic relationships as many of the participants lived with partners or spouses. | We agree with the reviewer that this is an interesting and important issue. We have added some data to further elaborate this finding (lines 253-257.  “For some participants, relationships with partners were often described as challenging, for example:  *I would imagine we’ve fallen out [wife’s name] and I, millions of times - she was going to leave me the next day if I didn’t change. Somehow we’ve managed to hang in there. (Mr M, 68, AS, AoD: 67)* |
|  | 5) In the theme ‘Health in the context of autism’, the authors focus mainly on coping with depression and stress. If indeed a lot of participants had co-occurring neurodevelopmental disorders, it would be interesting to read about their related experiences. This might be a good place to include the experiences of people with learning disabilities (or their relatives). Then, in the discussion, the authors write that the most common co-occurring mental health problem was anxiety, which was not reflected in the results, so perhaps elaborating on this would be helpful as well. | We have now clarified that the the majority of the sample overall, and all of the autistic adults interviewed directly, did not have co-occuring neurodevelopmental disorders, although we have now added some additional information about learning disability diagnoses where they applied (in addition to other new details described above). We have also provided further detail about the study participants, including information on the co-occurring diagnoses that participants provided in the cohort study surveys. Both depression and anxiety were commonly reported (n = 19 with depression; n = 15 with anxiety). The data we present and discuss in the paper reflects this – so we have removed reference to ‘and neurodevelopmental disorders’ from the ‘*health in context’* section of the results to avoid misrepresenting our findings. We have, also – in the discussion – revised the text to include both anxiety and depression as co-occuring health problems. |
|  | 6) The following part needs some correction: ‘Previously, ‘hiding difference’, was offered as a partial explanation for the difficulty identifying autism in girls and women. [citation needed] However there was little consideration of the burden on the individual nor that males and females might both struggle in this way [19].’ I think that the burden of camouflaging was recognized, including the study that the authors cite and some other studies. For example, camouflaging is known to correlate with depression in men (Lai et al., 2016). There is also at least one, large qualitative study on camouflaging in autistic adults that could be cited here (‘“Putting on My Best Normal”: Social Camouflaging in Adults with Autism Spectrum Conditions’ by Hull et al., 2017). | We thank the reviewer for these suggestions. We have revised and updated our references to the literature including the studies recommended by the reviewer. We have also extended the discussion to include some recent and emerging research that relates to our findings (see responses to R2 below).The revised discussion [see track changes throughouth] we feel provides a more substantive reflection on the study findings.  The two sentences highlighted by the reviewer have been removed. |
|  | Minor issues:  1) Interview topic guides should be included in the appendices. Knowing what questions were asked is important to put the results in context. | We agree with the reviewer and have now included the topic guides as appendices. |
|  | 2) The tables with participants' characteristics are rather lengthy, so I would shortly sum up the data in the main text as well (how many of the participants had each diagnosis, how many years passed since the diagnosis, their employment, and living status). | As well as extending Tables 1 and 2 to include more information about participant characteristics, we have summarised the data in the text, as recommended by the reviewer. |
|  | 3) It would be good to present the main themes and their summary descriptions in a table. | We agree. A table presenting the main themes, together with a brief descriptive summary of each, has now been added to the manuscript (Table 3; Line 284) |
|  | 4) GCSEs (line 287) – please provide an explanation for non-British readers. | An explanation has now been provided- *‘high school qualifications (General Certificate of Secondary Education (GCSE), typically undertaken aged 14-16 years’*  This is now explained with reference to the participant data provided in relation to educational attainment levels; before it appears in the quote referred to here. |
|  | 5) In different sections of the paper, the authors write that the study included participants ‘from across the UK’ or ‘from across England’ – please clarify that. | As noted above, further details are provided in the methods and the results sections. |
|  | Other points:  The underlying data (transcripts of the participants' accounts) were not made available, but I consider the authors' explanation sufficient. | Thank you, the understanding of our position on this is appreciated. |
| R2 | Reviewer #2: This is very good qualitative study. It deserves to be read widely. | We thank R2 for this comment. |
|  | As a practitioner and researcher working with this population most of the material and comment provided sounds familiar. So a key question is how original it is. | This is an important question. Our aim was to build on the existing literature by providing an opportunity using a qualitative research methodology with the adults and relatives of different ages [from early adulthood to older age] who had joined the ASC-UK, to share their opinions about their experiences in adult life including successes and challenges. With this in mind we provided a broad range of topics within the topic guides (now included with the manuscript). We believe that the findings complement, replicate and expand current understanding by providing qualitative information (i) from a wider age range of autistic adults; (ii) insights on successes as well as challenges and (iii) the complementary perspectives of the accounts from relatives describing the achievements and needs of the autistic adult known to them. This has been summarised at the end of the introduction (lines 118-124):  *This study will contribute to the existing literature in two key ways. Firstly, by undertaking a large and comprehensive study of the experiences of autistic adults spanning a wider age group than has been studied in qualitative research to date. Secondly, we approach the study of autistic adults’ experiences without presupposing what aspects of their lives and experiences are most important. Rather we invited participants to explore aspects of their lives that were important to them, from a broad range of topics.*  As such, we also argue that there are some specific, additional insights that come from our study:   - Some additional data/findings relating to how relatives of autistic adults see them experiencing particular challenges – and successes – in life - We provide – for the first time that we are aware of – some data on successes and positive achievements ‘in life’, from an older/more diverse age group of adults than previously studied |
|  | I agree there are few such qualitative studies on adults. There are a number of single case autobiographies. There are also likely to be chapters in books on autism in adulthood that reflect similar discourses (e.g. chapters 2 and 4 in Brugha T The Psychiatry of Adult Autism and Asperger Syndrome, 2018, OUP.)  Are the following similar studies considered:  A descriptive, qualitative study of the challenges that individuals with Autism Spectrum Disorder experience when transitioning from skills training programs into the open labor market in Cape Town, South Africa. Soeker MS. Work. 2020;65(4):733-747. doi: 10.3233/WOR-203127. PMID: 32310205  Communication and AAC in the lives of adults with autism: the stories of their older parents. Hines M, Balandin S, Togher L. Augment Altern Commun. 2011 Dec;27(4):256-66. doi: 10.3109/07434618.2011.587830. PMID: 22136364  Exploratory Study of Childbearing Experiences of Women With Asperger Syndrome. Gardner M, Suplee PD, Bloch J, Lecks K. Nurs Womens Health. 2016 Feb-Mar;20(1):28-37. doi: 10.1016/j.nwh.2015.12.001. Epub 2016 Feb 12. PMID: 26902438  and possibly: 'I definitely feel more in control of my life': The perspectives of young autistic people and their parents on emerging adulthood. Cribb S, Kenny L, Pellicano E. Autism. 2019 Oct;23(7):1765-1781. doi: 10.1177/1362361319830029. Epub 2019 Feb 28. | We thank the reviewer for this comprehensive set of suggestions. We have undertaken a full update of the relevant literature including the recommendations from both reviewers and some further studies that we have identified . We believe that the expanded and revised discussion section [see track changes] has improved the quality and utility of our manuscript. |
|  | Minor details and queries. The term Procedures should perhaps be Selection of participants or Participant sampling? | Thank you for this suggestion. We have revised the Methods section of the manuscript to include two categories: (1) participant recruitment (to cover those procedures) and (2) data collection, to cover the procedures relating to interviews. |
|  | Around 1% of the UK population has an autism diagnosis [1, 2]. This is not a correct reading of these two sources. And besides it is the population prevalence (the sources cited are correct) and not the number with a diagnosis (of which some will be false) that matter. | We have corrected this to:  It is estimated that around 1% of the UK population has autism [1, 2]. |
|  | For clarity it is more correct to claim that this is 'the largest qualitative study undertaken with a purposive sample of DIAGNOSED autistic adults living in a range of UK geographical locations with differing local services'. (a study limitation is that it does not include undiagnosed adults who may be the majority group). | We have corrected this in the strengths and limitations section, as suggested. |
|  | How many other countries have as in the UK set out to support reasonable adjustments for people with autism across multiple governmental responsibilities such as welfare, employment, housing, as well as education and health? (With reference to '... This study is an important wake-up call for policy makers, commissioners of services and service providers. In the UK autism specific legislation and clinical guidelines have been in place for the last decade to enable autistic adults to access the range of mainstream services they need. I...' | Although we appreciate that this is an important question, unfortunately it is beyond the scope of this current study. However we have added some further information to our conclusions acknowledging the importance of further work in these areas .  *This study is an important wake-up call for policy makers, commissioners of services and service providers both in the UK and internationally. In a recent international scoping review of diagnosis of autism in adulthood, the findings ‘suggest that receiving a diagnosis in adulthood has a significant emotional impact, but accessibility and processes are inconsistent, and formal support services are lacking’. Further the authors highlight the need for more research on autism diagnosis in adults especially adult with intellectual disability (Huang et al 2020).* |
